# Adenylosuccinic acid therapy ameliorates murine Duchenne Muscular Dystrophy

**DOI:** 10.1038/s41598-020-57610-w

**Published:** 2020-01-24

**Authors:** Cara A. Timpani, Craig A. Goodman, Christos G. Stathis, Jason D. White, Kamel Mamchaoui, Gillian Butler-Browne, Nuri Gueven, Alan Hayes, Emma Rybalka

**Affiliations:** 1 Institute for Health and Sport, Victoria University, Melbourne, Victoria 8001 Australia; 2 Australian Institute for Musculoskeletal Science (AIMSS), Victoria University, St Albans, Victoria 3021 Australia; 3 Murdoch Children’s Research Institute, Royal Children’s Hospital, Parkville, Victoria Australia; 4 Melbourne Veterinary School, University of Melbourne, Parkville, Victoria Australia; 5 Institut de Myologie, Sorbonne University, INSERM UMRS974, Paris, France; 6 Pharmacy, School of Medicine, University of Tasmania, Hobart, Tasmania 7000 Australia; 7 Department of Medicine-Western Health, The University of Melbourne, St Albans, Victoria 3021 Australia

**Keywords:** Neuromuscular disease, Preclinical research

## Abstract

Arising from the ablation of the cytoskeletal protein dystrophin, Duchenne Muscular Dystrophy (DMD) is a debilitating and fatal skeletal muscle wasting disease underpinned by metabolic insufficiency. The inability to facilitate adequate energy production may impede calcium (Ca^2+^) buffering within, and the regenerative capacity of, dystrophic muscle. Therefore, increasing the metabogenic potential could represent an effective treatment avenue. The aim of our study was to determine the efficacy of adenylosuccinic acid (ASA), a purine nucleotide cycle metabolite, to stimulate metabolism and buffer skeletal muscle damage in the *mdx* mouse model of DMD. Dystrophin-positive control (C57BL/10) and dystrophin-deficient *mdx* mice were treated with ASA (3000 µg.mL^−1^) in drinking water. Following the 8-week treatment period, metabolism, mitochondrial density, viability and superoxide (O_2_^−^) production, as well as skeletal muscle histopathology, were assessed. ASA treatment significantly improved the histopathological features of murine DMD by reducing damage area, the number of centronucleated fibres, lipid accumulation, connective tissue infiltration and Ca^2+^ content of *mdx* tibialis anterior. These effects were independent of upregulated utrophin expression in the tibialis anterior. ASA treatment also increased mitochondrial viability in *mdx* flexor digitorum brevis fibres and concomitantly reduced O_2_^−^ production, an effect that was also observed in cultured immortalised human DMD myoblasts. Our data indicates that ASA has a protective effect on *mdx* skeletal muscles.

## Introduction

Characterised by progressive and fatal muscular weakness and degeneration, Duchenne Muscular Dystrophy (DMD) is a rare neuromuscular disorder that arises from the loss of dystrophin at the sarcolemma due to a genetic defect in the encoding gene^1^. It is generally accepted that the pathophysiology of DMD is induced by calcium (Ca^2+^) dysregulation secondary to dystrophin deficiency^2–7^ which leads to activation of Ca^2+^-dependent enzymes^8–11^ and the progression of muscle damage, degeneration and wasting, and chronic inflammation^12^. As muscle is replaced with fatty and/or fibrous connective tissue, weakness ensues. Consequently, DMD sufferers are typically wheelchair bound by early adolescence^13^ and die from cardiorespiratory failure before thirty years of age^13^. One often unaddressed characteristic of dystrophin-deficient muscle is associated metabolic dysfunction, which is evident across various metabolic pathways responsible for cellular energy production (as reviewed in^14^). While this metabolic dysfunction is often regarded as a secondary consequence of Ca^2^^+^-dependent disease sequelae, we hypothesised that mitochondrial dysfunction in particular is a core aetiological perturbation^14^ that could be therapeutically exploited. Together with: (1) the initial observations of slower running speeds in *mdx* mice^15^; (2) the observations that metabolic dysfunction is present in dystrophic myoblasts prior to the normal expression of dystrophin protein^16^; (3) the observation that mitochondrial adenosine triphosphate (ATP) production rate is reduced in isolated dystrophic mitochondria removed from the dystrophic pathological environment and bathed in the presence of an optimal extracellular environment^17^; and (4) positive clinical trials data with the mitochondrial short chain CoQ10 analogue, idebenone, in DMD patients^18^; our hypothesis of metabolic dysfunction as an aetiological driver of DMD has been given credence.

During the extensive investigation of metabolic therapies to treat DMD in the 1980–90’s (as reviewed by us previously^19^), one promising therapeutic was the purine nucleotide, adenylosuccinic acid (ASA), which was investigated in a long term, Phase I clinical trial including both Duchenne and Becker (a milder variation) MD patients^20^. ASA is a metabolite of the Purine Nucleotide Cycle (PNC), which is activated during metabolic stress to drive the recovery of ATP from inosine monophosphate (IMP) via the reversible reaction: IMP → AMP → ADP → ATP^20^. The PNC also produces fumarate that can be shuttled into the mitochondria to anaplerotically expand the Tricarboxylic (citric) Acid (TCA) cycle and, therefore, enhance ATP production capacity^20^. Following ASA administration, patients anecdotally reported instantaneous increases in energy, stamina and endurance^20^. Functionally, ASA therapy maintained the ability to stand erect, rise from the floor and walk without falling, which was accompanied with decreased serum creatine kinase (CK) levels and improvements in histopathological hallmarks indicating a reduction in muscle damage^20^. The subsequent replacement of functional muscle with fatty and connective tissue is a feature of disease progression and leads to reduced physical capacity^21–23^. In support of ASA-induced protection, significantly reduced fatty tissue infiltration was observed in muscle biopsies taken at multiple time points during the four-year trial. The ability of ASA to improve key features of DMD, including the maintenance of muscle function, may arise from its capacity to promote ATP production by increased purine salvage and aerobic metabolism via purine nucleotide cycling and anaplerotic expansion of the TCA cycle, respectively. Recently, it has been demonstrated that ASA stimulates exocytosis of insulin from pancreatic β cells, and that inhibition of ASA’s regulatory enzymes within the PNC impairs glucose-stimulated insulin secretion^24^. This suggests that ASA activates energy producing pathways, which could be beneficial to overcome the chronic metabolic impairment of dystrophin-deficient muscles^17,25–31^.

A major limitation of the clinical ASA trial is that only one DMD patient completed the study long-term. ASA was beneficial in two BMD patients, but its long-term capacity to affect the progression of DMD has never been determined. Thus, this proof-of-concept study aimed to determine the therapeutic potential of ASA for the treatment of DMD. We investigated the effects of 8 weeks of ASA therapy in healthy (control; CON) and dystrophic (*mdx*) mice, and specifically assessed whether ASA supplementation could attenuate the histopathological progression of DMD by improving bioenergetical status and mitochondrial capacity of skeletal muscle. We hypothesised that ASA therapy would: (1) reduce the histopathological hallmarks of DMD such as muscle damage and lipid and connective tissue infiltration; and (2) improve mitochondrial function and the overall bioenergetical capacity of skeletal muscles in *mdx* mice.

## Materials and Methods

### Ethical approval

All experimental procedures were approved by the Victoria University Animal Ethics Committee and conformed to the Australian Code of Practice for the Care and Use of Animals for Scientific Purposes.

### Animals and treatment

Three-week old male C57Bl/10ScSn (normal wild-type strain; CON) and C57Bl/10*mdx* (*mdx*) mice were purchased from Animal Resources Centre (Western Australia, Australia) and housed at the Western Centre for Health, Research and Education (Sunshine Hospital, Victoria, Australia) on a 12:12 hour light-dark cycle with *ad libitum* access to food and water. Mice of the same strain were randomly assigned to housing cages of four by animal technicians on arrival. At four weeks of age, cages were randomly block assigned into untreated (CON and *mdx*) and treated (CON ASA and *mdx* ASA) groups. For the ASA treated groups, we aimed to administer a human equivalent dose of 25 mg/kg/day as this is the only specified dose that was delivered via a non-intravenous route during the clinical trial of ASA^20^. After taking into account blood volume and the average daily water intake of a mouse, mice were administered 3000 µg/mL ASA in RO drinking water (pH 7.2). A progressive treatment protocol was employed to enable the detection of any toxic or adverse effects in mice. Mice were initially administered 3 µg/mL ASA for 3 days and this was increased to 30 µg/mL for the next 4 days, and 300 µg/mL of ASA for one week. Since no adverse effects were observed, 3000 µg/mL ASA was delivered for the remaining 6 weeks of the treatment period. Based upon cage water consumption, the average daily exposure of mice during the first 7 days was 2.99 ± 0.10 mg/kg/day for CON mice and 3.04 ± 0.19 mg/kg/day for *mdx* mice. In the second week, the daily exposure of mice was 35.89 ± 2.48 mg/kg/day for CON mice and 40.20 ± 2.20 mg/kg/day for *mdx* mice. The daily exposure for the final 6 weeks of treatment was 325.74 ± 11.43 mg/kg/day for CON mice and 335.64 ± 50.42 mg/kg/day for *mdx* mice. Using a conversion coefficient recommended by the US Food and Drug Administration of 12.3 to account for differences in body surface area between humans and mice^32^, the estimated daily human equivalent dosage was ~26 mg/kg/day for CON ASA mice and ~27 mg/kg/day for *mdx* ASA mice. This approximates the human equivalent target dosage of 25 mg/kg/day in the ASA clinical trials^20^.

At the conclusion of the treatment period, mice were deeply anaesthetised (intraperitoneal injection of 60 mg/kg sodium pentobarbitone) and non-survival surgery was performed. Skeletal muscles were removed for analyses in the following order: (1) left and right flexor digitorum brevis (FDB) for the measurement of mitochondrial parameters; (2) left and right extensor digitorum brevis (EDL) and soleus for the assessment of contractile properties; (3) right and left tibialis anterior (TA) for the analysis of histopathology and metabolites, respectively; and (4) right and left quadriceps for western blot analyses of proteins of interest. The remaining hind limb skeletal muscles, diaphragm and organs (including the heart, lungs, liver and spleen) were also surgically excised and weighed.

### Histopathology

The right TA was covered in optimal cutting temperature compound (Sakura Finetek) and snap frozen in liquid nitrogen-cooled isopentane. TA’s were sectioned on a cryostat (10 µm, -20 °C, Leica CM1950) and mounted onto glass slides (Menzel-Glaser).

Five histological stains were employed to assess various features of the dystrophic histopathology. Haematoxylin & Eosin (H&E) was utilised to assess fibre size, damage area (measured as areas of myofibres dissolution with inflammatory/satellite cell infiltrate^33^) and centronucleated fibres, while Oil Red O (ORO) evaluated lipid accumulation within the whole muscle. Collagen deposition, which increases as the disease progresses, was evaluated via Gomori Trichrome staining (HT10316, Sigma Aldrich) with relative Ca^2+^ content assessed via Alizarin Red staining (Merck Millipore). Finally, succinate dehydrogenase (SDH) activity was measured via histochemical analysis to determine any differences in oxidative capacity of the TA^34^. All histological protocols were performed as described previously^35,36^. For H&E and ORO, slides were imaged on a microscope (Zeiss Axio Imager Z2) at 20x and 10x magnification, respectively. For Gomori Trichrome, Alizarin Red and SDH stains, slides were imaged on a microscope (Olympus, Tokyo, Japan) at 40x magnification. All images were analysed using ImageJ software (NIH, USA) as previously described^36,37^.

### Contractile properties

Muscle (EDL and soleus) dissection and preparation, and the contraction protocol was performed as previously described^37^. Optimal length (L_o_) was determined via a series of twitch contractions, and the left EDL and SOL were stimulated to contract tetanically (maximal activation at 100 Hz) to obtain absolute force (P_o_). Muscles were then blotted dry and weighed and the cross-sectional area (CSA) was determined (CSA = muscle mass (g)/(L_o_ (cm)*[fibre length/muscle length])*density. For EDL and soleus, the fibre length/muscle length ratio was 0.44 and 0.71, respectively, and the density was 1.06 g/cm_2_ for both muscles^38^. Specific force (sP_o_) for each muscle was calculated sP_o_ = (Po/1000)/CSA.

### Mitochondrial respiration measurement

Mitochondrial respiratory parameters were quantified in isolated muscle fibres as described previously by Schuh *et al*.^39^. Left and right FDB were excised from anaesthetised mice and incubated in pre-warmed dissociation media (DMEM, Gibco, 10566016; 2% FBS, Bovogen Biologicals; 4 mg/mL collagenase A, Roche, 10103586001; 50 µg/mL gentamycin, Sigma Aldrich, G1397) for 1 hour and 45 minutes (37 °C, 5% CO_2_). Following the dissociation period, FDB bundles were placed into incubation media (DMEM, Gibco, 10566016; 2% FBS, Bovogen Biologicals; 50 µg/mL gentamycin, Sigma Aldrich G1397) and bundles were triturated with graduated pipette tips to yield single fibres. To facilitate fibre adherence to Seahorse XF24 cell culture V7 microplates (Seahorse Bioscience, MA, USA), all wells of the microplate were coated with extracellular matrix (Sigma Aldrich, E1270) and a 75 µL aliquot of isolated fibres were placed into the coated wells (all samples were run in triplicate). Confluency (~60%) was determined using a light microscope. Following overnight incubation, incubation media was replaced with pre-warmed measurement buffer (120 mM NaCl, 3.5 mM KCl, 1.3 mM CaCl_2_, 0.4 mM KH_2_PO_4_, 1 mM MgCl_2_, 5 mM HEPES, 2.5 mM D-glucose and 0.5mM L-carnitine, pH 7.4) and the microplate was re-incubated for 2 hours for pH and temperature equilibration. Oxygen consumption rate (OCR) and extracellular acidification rate (ECAR) were determined for various respiratory states using a mitochondrial stress test protocol. A loaded Sensor Cartridge (all final concentrations in the well following injection: Port A: 2 µg/mL oligomycin; Port B: 400 nM FCCP and 10 mM pyruvate; Port C: 1 µM antimycin A) was inserted into the Seahorse Bioscience XF24 Analyser and once calibration was completed, the sample loaded microplate was inserted. Following an equilibration period, basal OCR and ECAR were measured with a 3 minute mix, 2 minute wait, and 3 minute measure cycle, which was looped 3 times. Port A was injected to induce state 4 respiration and following the 3 mix-wait-measure cycles, Port B was injected to induce state 3 respiration. Following the subsequent 3 mix-wait-measure cycles, Port C was injected to inhibit respiration and detect non-mitochondrial respiration.

### Mitochondrial density, viability and superoxide (O_2_^−^) production

Mitochondrial viability in isolated FDB fibres was assessed by the fluorescent MitoTracker dyes Green and Red. MitoTracker Green is a non-selective mitochondrial dye that labels all mitochondria irrespective of the mitochondrial membrane potential (∆Ψ) while MitoTracker Red is only taken up into mitochondria with a ∆Ψ. Isolated FDB fibres were plated onto a matrigel coated 96 well microplate and confluency was determined using a light microscope. The microplate was incubated overnight and 10 minutes prior to the addition of the MitoTracker dyes, FCCP and antimycin A (final concentration of 3 µM each) were added to positive control wells to induce mitochondrial death. Following this, a cocktail of MitoTracker Green and Red (final concentration of 200 nM and 50 nM, respectively) was added to each well and incubated at 37 °C for 30 minutes. Fibres were then washed twice with Fluorobrite media and imaged on an inverted microscope (Olympus, Tokyo, Japan) using FITC and TRITC filters. Images were analysed using ImageJ software (NIH, USA) and mitochondrial viability was calculated as a ratio of live mitochondria (MitoTracker Red) to total mitochondrial pool (MitoTracker Green).

Mitochondrial O_2_^−^ production was measured as described by us previously^36^, using the O_2_^−^ indicator MitoSOX Red, which is selective to mitochondria and fluoresces red when oxidised by mitochondrial O_2_^−^. Isolated FDB fibres were plated into matrigel-coated wells in triplicate and confluency was determined using a light microscope. Plates were incubated overnight and 5 minutes prior to the addition of the MitoTracker dyes, antimycin A (final concentration of 3 µM) was added to the positive control wells, which inhibits Complex III (CIII) and drives reverse electron flow and maximal O_2_^−^ production at Complex I (CI). Following this, MitoSOX (final concentration of 5 µM) in HBSS/Ca^2+^/Mg^2+^ buffer (10 mM HEPES, 150 mM NaCl, 5 mM KCl, 1 mM MgCl, 1.8 mM CaCl_2_, pH 7.4) was added to wells and plates were re-incubated at 37 °C for 30 minutes. MitoSOX was removed and fibres were counterstained with MitoTracker Green. Fibres were then washed twice with Fluorobrite media and imaged as above.

### Western blot analysis of metabolic stress (AMPK), mitochondrial biogenesis (PGC-1α/β), respiratory chain proteins (CI-V) and utrophin

Western blot analysis of metabolic stress, mitochondrial biogenesis, respiratory chain proteins and utrophin was performed as previously described^35^. Briefly, frozen quadriceps (for AMPK, PGC-1α/β and CI-V) and TA (utrophin) were homogenised in ice-cold WB buffer (40 mM Tris, pH 7.5; 1 mM EDTA; 5 mM EGTA; 0.5% Triton X-100; 25 mM β-glycerophosphate; 25 mM NaF; 1 mM Na3VO4; 10 μg/ml leupeptin; and 1 mM PMSF), and protein concentrations were determined (DC protein assay kit, Bio-Rad Laboratories, Hercules, CA, USA). 15 μg of protein from each sample was separated on SDS-PAGE acrylamide gels and once complete, transferred to a PVDF membrane. After blocking with 5% powdered milk, membranes were incubated overnight at 4 °C with the primary antibody Total OXPHOS Antibody Cocktail (1:1000, mouse, Abcam, #ab110413; Total AMPK-α (1:1000, rabbit, Cell Signalling, #2603 S); Phospho-AMPK Thr^172^ (1:1000, rabbit, Cell Signalling, #2535 s); PGC-1α (1:1000, mouse Merck Millipore, #ST1202); PGC-1β (1:3000, rabbit, Abcam, #ab176328); utrophin (1:200, mouse, Developmental Studies Hybridoma Bank (MANCH03 (8A4)-c; deposited by Morris, G.E.)). After overnight incubation, the membranes were washed, incubated with a peroxidase-conjugated secondary antibody (1:20,000, anti-mouse, #PI-2000; 1:5000, anti-rabbit, #PI-1000; Vector Labs) at room temperature and washed again. Images were captured (Fusion FX imaging system, Vilber Lourmat, Germany) once the blots were developed with ECL Prime reagent (Amersham, Piscataway, NJ, USA). Densitometric analysis was performed using Fusion CAPT Advance software (Vilber Lourmat, Germany). Membranes were then stained for total protein with Coomassie Blue^40^. The signal for the band of the protein of interest was then normalized to the signal for total protein in each lane.

### Citrate synthase (CS) activity

CS activity was measured as a marker of mitochondrial density and/or anaplerosis^41^. Homogenised FDB fibres were added to the reagent cocktail (100 mM Tris Buffer, 1 mM DTNB, 3 mM Acetyl CoA) and to initiate the reaction, oxaloacetate (10 mM) was added just prior to measuring CS activity spectrophotometrically (412 nm, 25 °C, 5 mins). CS activity was calculated using the extinction coefficient of 13.6^42^.

### Metabolite quantification

ATP, phosphocreatine (PCr), creatine (Cr), lactate and glycogen metabolites were assessed in the left TA. At least 20 mg of frozen sample was weighed and freeze-dried at −40 °C (Edwards Modulo, Edwards High Vacuum, Britain, England) for a minimum of 48 hours. For ATP, PCr, Cr and lactate metabolite extraction, 2 mg of powdered sample was used while 1 mg of powdered was utilised for glycogen metabolite extraction. Metabolite analysis was performed in 96 well plates using a method adapted from Lowry and Passonneau^43^ as described by us previously^44^.

### Statistics

Data are presented as mean ± standard error of the mean. A two-way ANOVA was utilised to detect genotype and treatment differences. When a main effect or an interaction was detected, unpaired T-tests were used to determine differences between individual groups using SPSS (version 21). An α value of 0.05 was considered significant.

## Results

### Effect of ASA on body weight, food and water consumption and muscle and organ weights

Throughout the 8-week treatment period, weight gain (% of pre-treatment body weight) was comparable between CON and *mdx* strains both with and without ASA treatment (Fig. S1A). However, post-treatment body weights were different, with *mdx* mice being heavier compared to CON mice (*p* < 0.01, Table 1). ASA reduced the post-treatment body weight of *mdx* (*p* < 0.05, Table 1) but not CON mice, and this effect was independent of food and water consumption which was comparable between strain and treatment protocols (Fig. S1B,C). Hind limb muscle weights (EDL, gastrocnemius, quadriceps, SOL and TA) relative to body weight were heavier in *mdx* compared to CON mice (*p* < 0.01–0.0001; Table 1), except for *mdx* plantaris weights, which were comparable to CON. ASA had no effect on muscle weights in either strain, except for an anomaly reduction in the right CON TA which was not evident in the left CON TA (*p* < 0.05).

**Table 1:** Post treatment body weights and muscle and organ weights relative to the body weight of untreated and ASA-treated mice.

	CON		*mdx*	
	UNTREATED	ASA	UNTREATED	ASA
Body weight (final, g)	30.2 ± 0.4	30.9 ± 0.4	32.4 ± 0.5^^	31.3 ± 0.5^^ *
Diaphragm (mg/g bw)	2.76 ± 0.14	2.33 ± 0.21	3.37 ± 0.14^^	3.53 ± 0.17
EDL (L, mg/g bw)	0.4 ± 0.02	0.42 ± 0.02	0.54 ± 0.02^^^^	0.52 ± 0.03
EDL (R, mg/g bw)	0.38 ± 0.03	0.40 ± 0.01	0.53 ± 0.03^^^^	0.50 ± 0.02
Gastrocnemius (L, mg/g bw)	4.84 ± 0.24	4.60 ± 0.13	5.66 ± 0.18^^	5.59 ± 0.15
Gastrocnemius (R, mg/g bw/g bw)	5.07 ± 0.14	5.02 ± 0.16	5.97 ± 0.26^^	5.67 ± 0.10
Heart (mg/ g bw)	4.76 ± 0.11	4.65 ± 0.09	4.40 ± 0.07^^	4.28 ± 0.07
Liver (mg/g bw)	49.57 ± 1.36	47.85 ± 3.85	55.52 ± 1.06^	55.12 ± 1.46
Lungs (mg/g bw)	6.74 ± 0.30	5.92 ± 0.24*	6.05 ± 0.19	5.68 ± 0.20
Plantaris (L, mg/g bw)	0.63 ± 0.04	0.65 ± 0.04	0.69 ± 0.03	0.70 ± 0.04
Plantaris (R, mg/g bw)	0.68 ± 0.08	0.62 ± 0.04	0.71 ± 0.02	0.70 ± 0.03
Quadriceps (L, mg/g bw)	6.5 ± 0.36	6.44 ± 0.22	8.97 ± 0.26^^^^	8.95 ± 0.24
Quadriceps (R, mg/g bw)	6.24 0.34	5.92 0.27	8.16 0.21^^^^	8.40 0.29
Soleus (L, mg/g bw)	0.37 ± 0.02	0.38 ± 0.02	0.48 ± 0.02^^^^	0.50 ± 0.02
Soleus (R, mg/g bw)	0.35 ± 0.02	0.37 ± 0.02	0.44 ± 0.02^^^	0.47 ± 0.03
Spleen (mg/g bw)	3.9 ± 0.1	4.0 ± 0.1	3.9 ± 0.2	3.8 ± 0.1
TA (L, mg/g bw)	1.6 ± 0.10	1.61 ± 0.08	2.30 ± 0.08^^^^	2.23 ± 0.06
TA (R, mg/g bw)	1.87 ± 0.11	1.60 ± 0.05*	2.24 ± 0.05^^^	2.23 ± 0.06

Final body weights were significantly higher in *mdx* compared to CON mice (*p* < 0.01) with ASA reducing the body weight of *mdx* mice (*p* < 0.05). Relative to body weight, the weights of all skeletal muscles and organs were significantly higher in *mdx* compared to CON mice (*p* < 0.05), with the exception of the lungs, spleen and left and right plantaris which were comparable between genotypes (*p* > 0.05). ASA had no effect on the weight (mg/g bw) of any *mdx* tissues, but it did reduce the lung and right TA weight (mg/ g bw) in CON mice (*p* < 0.05). ^*p* < 0.05, ^^*p* < 0.01, ^^^*p* < 0.005, ^^^^*p* < 0.001 *mdx* significantly different from CON mice; *p < 0.05 ASA-treated significantly different from untreated *n* = 7–14 CON, *n* = 11–16 CON ASA, *n* = 10–16 *mdx*, *n* = 11–16 *mdx* ASA.^95^.

Since DMD boys die from cardiorespiratory insufficiency, we also measured heart, diaphragm and lung weights to determine any effects of genotype or ASA treatment on the cardiorespiratory system. While the diaphragm weight relative to body weight was higher in *mdx* compared to CON mice (*p* < 0.01) as per the hind limb skeletal muscles, the heart weight relative to body mass was reduced in *mdx* compared to CON mice (*p* < 0.01) and there was a strong trend for a similar genotype-dependent reduction in lung weight (*p* = 0.060). ASA reduced the lung weight in CON (*p* < 0.05) but not *mdx* mice and had no effect on the mass of the diaphragm or any other organ assessed.

### ASA ameliorates histopathology

#### ASA reduces pseudohypertrophy and damage in mdx TA

**Figure 1:**
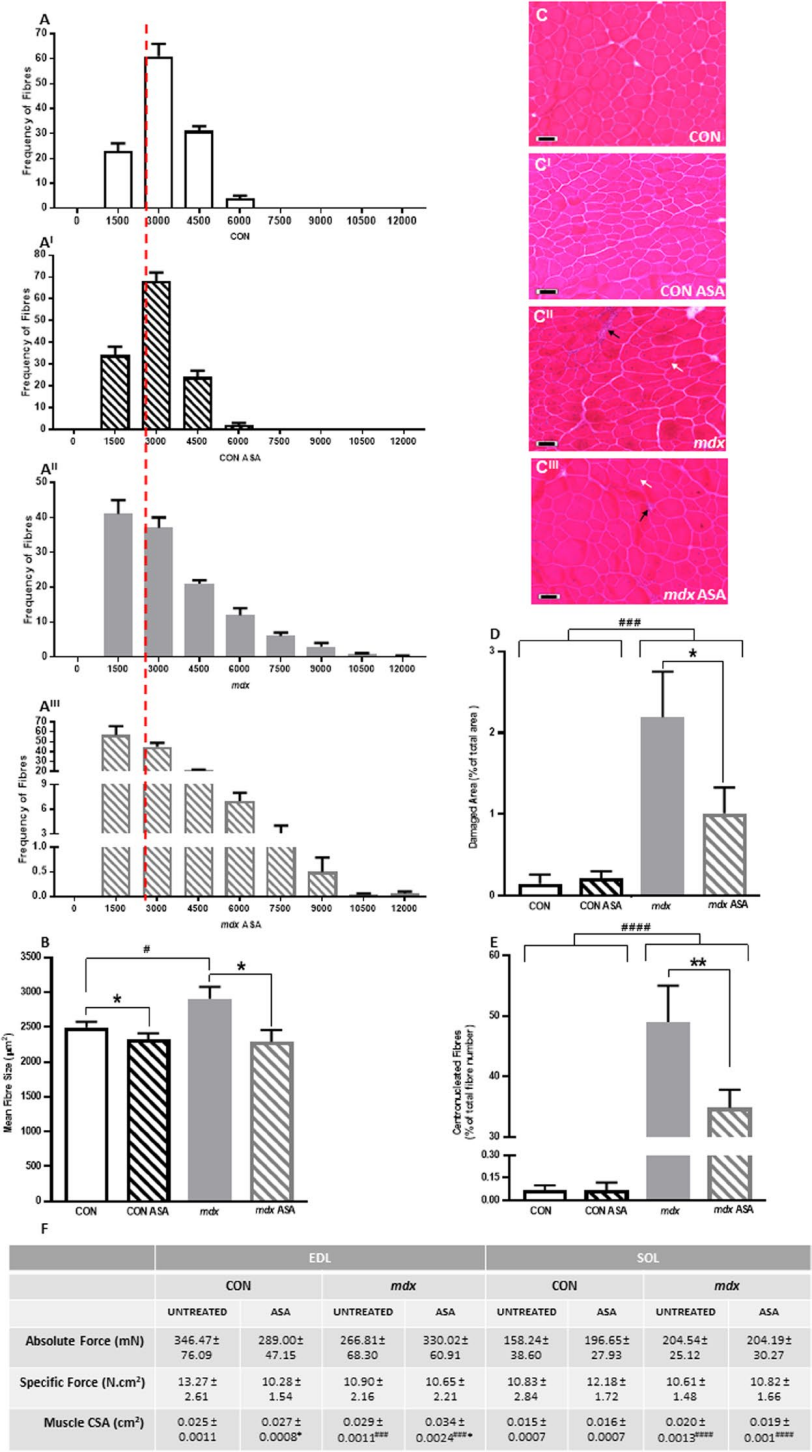
ASA attenuates damage and pseudohypertrophy of *mdx* tibialis anterior (TA) but has no effect on contractile properties of the extensor digitorum longus (EDL) or soleus (SOL). The frequency histogram of CON (**A**), CON ASA (A^I^), *mdx* (A^II^) and *mdx* ASA (A^III^) shows the shift in histogram shape. The red dotted line indicates the mean fibre size of CON TA fibres. Mean fibre size was larger in *mdx* compared to CON TA (*p* < 0.05, (**B**)) with ASA decreasing mean fibre size in both CON and *mdx* fibres (*p* < 0.05). Re*p*resentative images of *mdx* (C^II^) and *mdx* ASA (C^III^) TA cross sections indicate damaged areas (black arrows) and centronucleated fibres (white arrows) which are not evident in CON (**C**) and CON ASA (C^I^) TA. Damaged area (**D**) and percentage of centronucleated fibres (**E**) was significantly higher in *mdx* TA compared to CON (*p* < 0.001 and *p* < 0.0001 respectively) with ASA decreasing damage and regeneration in *mdx* TA (*p* < 0.05 and *p* < 0.01, respectively). No significant differences in absolute or specific force (F) were observed in the EDL or SOL of any group. The muscle cross sectional area (CSA) was larger in *mdx* EDL (*p* < 0.001) and SOL (*p* < 0.0001) compared to CON. ASA increased the CSA of both CON and *mdx* EDL (*p* < 0.05). For histology: *n* = 9 CON, *n* = 9 CON ASA, *n* = 10 *mdx*, *n* = 11 *mdx* ASA. For EDL contractile: *n* = 11 CON, *n* = 15 CON ASA, *n* = 5–6 *mdx*, *n* = 13–15 *mdx* ASA. For SOL co*n*tractile: *n* = 9–12 CON, *n* = 12 CON ASA, *n* = 6 *mdx*, *n* = 13–14 *mdx* ASA. Scale bar = 50 μm.

The mean fibre CSA was 17% greater in untreated *mdx* compared to CON TA (*p* < 0.05, Fig. 1C), which was associated with a shift in fibre size distribution to the right due to there being a higher frequency of fibres with a larger CSA (6000–13499 μm^2^, *p* < 0.0001, Fig. 1A). ASA treatment reduced the mean fibre size in both CON and *mdx* TA by 7% and 21%, respectively (*p* < 0.05, Fig. 1C). In particular, a decrease in the number of fibres with a CSA between 6000 and 13499 μm^2^ was observed in *mdx* ASA-treated TA (*p* < 0.05, Fig. 1A), which shifted the fibre size distribution to the left (Fig. 1A). As expected, the proportional area of damage was significantly higher in *mdx* compared to CON TA (*p* < 0.001; Fig. 1D). While the damaged area represented only ~2% of the total CSA (which is consistent with the stabilisation phase characteristic of 12w old *mdx* mice), ASA remarkably reduced the damaged area by 46% in *mdx* TA (*p* < 0.05, Fig. 1D). In ASA-treated *mdx* TA, the reduction in damaged area corresponded with a reduction (of 29%) in centronucleated fibres (an indicator of muscle regeneration) compared to untreated *mdx* TA (*p* < 0.01, Fig. 1E).

#### ASA reduces lipid content of mdx TA

**Figure 2:**
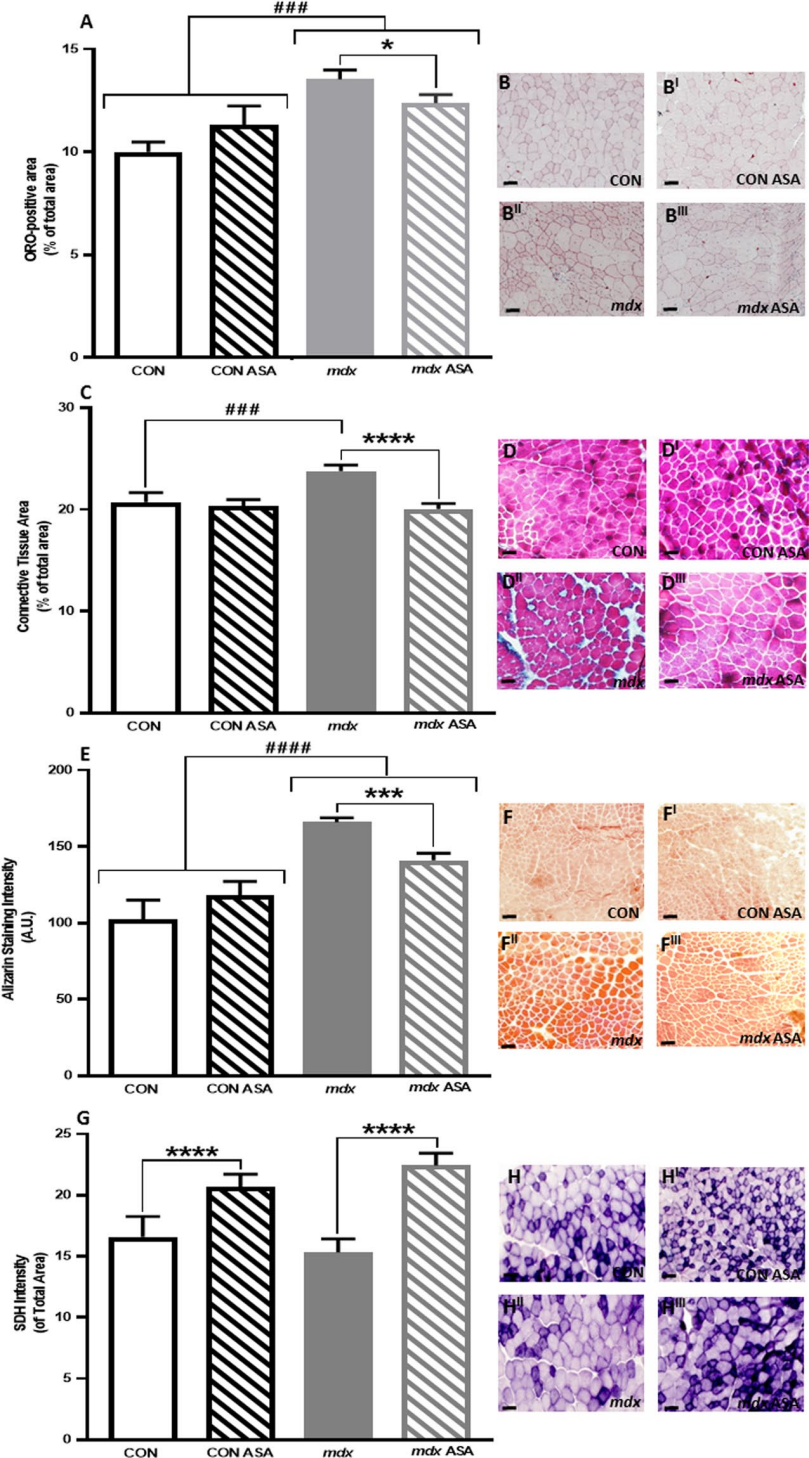
Assessment of lipid accumulation (ORO), connective tissues (Gomori), Ca^2+^ content (Alizarin Red) and succinate dehydrogenase (SDH) content in TA from untreated and ASA-treated CON and *mdx* mice. Oil Red O (ORO) positive area of *mdx* TA was significantly higher compared to CON (*p* < 0.001, **A**) with ASA reducing ORO positive area of *mdx* TA compared to *mdx* UNSUPP (*p* < 0.05). Connective tissue was also significantly higher in *mdx* TA compared to CON (*p* < 0.001, **C**) with ASA reducing the connective tissue content of *mdx* TA (*p* < 0.0001). Ca^2+^ content was significantly higher in *mdx* TA compared to CON (*p* < 0.0001, **E**) with ASA reducing Ca^2+^ content in *mdx* TA (*p* < 0.001). The SDH positive area was comparable between CON and *mdx* TA (*p* > 0.05, **G**) with ASA increasing the SDH positive area in both CON ASA and *mdx* ASA TA (*p* < 0.0001). *n* = 8–12 CON, *n* = 10–12 CON ASA, *n* = 9–12 *mdx*, *n* = 10–12 *mdx* ASA. Scale bar = 50 μm.

Previously it has been demonstrated that ASA reduces lipid production in DMD muscle explants^45^. As such, we assessed the effect of ASA on the lipid content of dystrophic muscle. Neutral lipids were quantified in whole cross-sections to give the overall ORO positive area. In *mdx* TA, the ORO positive area was 34% greater compared to CON TA (*p* < 0.001, Fig. 2A). ASA significantly reduced the ORO positive area by ~10% in *mdx* TA (*p* < 0.05).

#### ASA reduces the collagen content of mdx TA

Next, we assessed the effect of ASA on the connective tissue content of dystrophic muscle, since connective tissue accumulation and fibrosis is a feature of disease progression. In untreated *mdx* TA, connective/fibrotic tissue abundance was 15% greater compared to CON TA (*p* < 0.001, Fig. 2C). Remarkably, ASA normalised the connective/fibrotic tissue content in *mdx* TA to CON levels (*p* < 0.0001).

#### ASA reduces Ca^2+^ content of mdx TA

Since Ca^2+^ dysregulation is a well-documented consequence of dystrophin-deficiency and is a driver of the pathological muscle degeneration in DMD, we investigated whether ASA could positively modulate this important pathological feature. Intramuscular Ca^2+^ content, as assessed by the staining intensity of Alizarin Red, was 62% higher in untreated *mdx* compared to CON TA (*p* < 0.0001, Fig. 2E). Although, ASA had no effect on the Ca^2+^ content of CON TA (*p* > 0.05, Fig. 2E), ASA treatment significantly reduced the Ca^2+^ content of *mdx* TA by 15% (*p* < 0.001).

#### ASA increases SDH in mdx TA

SDH, a mitochondrial enzyme shared by the TCA cycle and the electron transport chain (ETC), is a marker of oxidative fibre type with deep purple fibres being characteristic of Type I fibres and lighter purple fibres being indicative of Type IIa fibres. Assessing the whole TA cross-sectional area for SDH staining indicates the total mitochondrial content of all fibre types. While there was no difference in SDH positive area between untreated CON and *mdx* TA (Fig. 2G), ASA treatment did increase the SDH positive area of both CON and *mdx* TA by 25% and 46%, respectively (*p* < 0.0001, Fig. 2G). This suggests a capacity to regulate mitochondrial content/density.

### ASA has no effect on muscle contractile properties

A distinguishing feature of DMD progression is the replacement of functional skeletal muscle with non-functional fatty and connective tissue which results in reduced force production and myofibril pseudohypertrophy. Thus, we assessed contractile properties in the fast-twitch EDL and slow-twitch soleus muscle. There was no significant difference in P_o_ between CON and *mdx* muscles or following ASA treatment (Fig. 1F). Despite an increase in CSA in both *mdx* EDL (*p* < 0.001, Fig. 1F) and *mdx* SOL compared to CON (*p* < 0.0001, Fig. 1F), the sP_o_ of *mdx* EDL and SOL was still comparable (Fig. 1F), albeit *mdx* EDL were ~20% lower than CON. These data are somewhat consistent with a higher non-functional tissue content of fast-twitch muscles as demonstrated histologically in the TA. In both CON and *mdx* EDL, ASA increased the CSA compared to CON (Fig. 1F), however this effect was not observed in SOL (Fig. 1F) and had no effect on sPo.

### Effects of ASA treatment on mitochondrial and metabolic parameters

#### Respirometry

Considering the significant improvements in energy and stamina reported by patients in the ASA clinical trial^20^, and the capacity of purine nucleotide cycling to both generate fumarate for anaplerosis and potentiate adenine nucleotide salvage, we investigated whether the improvements in muscle histopathology might be due to improved mitochondrial function. As we could not reliably quantify protein content of the wells to internally correct respiration values for mitochondrial density due to very low protein concentrations, here we present respiration parameters that are internally corrected for the basal respiration.

**Figure 3:**
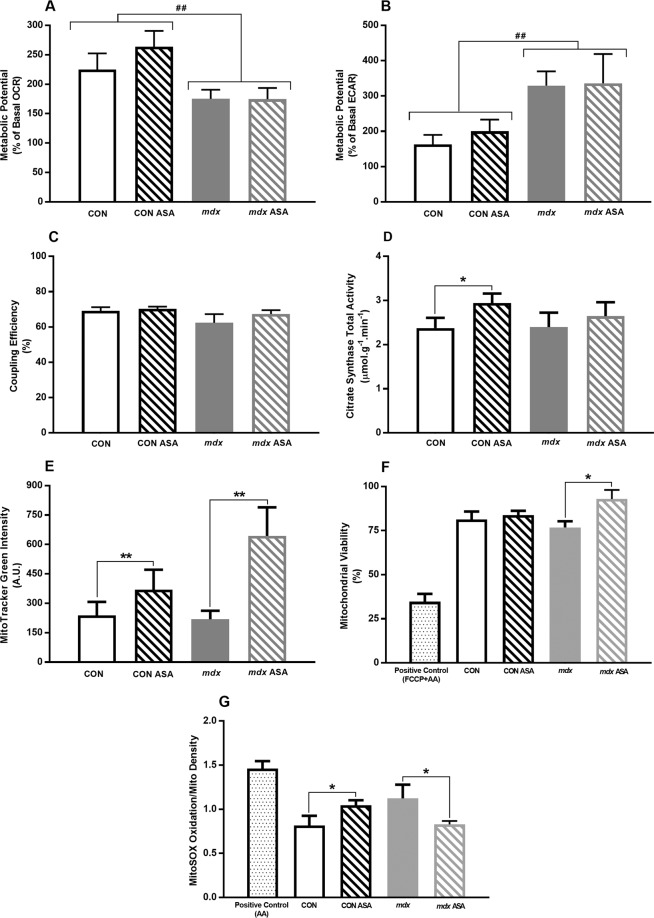
Mitochondrial function, viability, density and superoxide (O_2_^−^) production in isolated FDB fibres from untreated and ASA-treated CON and *mdx* mice. The oxidative metabolic potential was significantly less in *mdx* compared to CON FDB fibres (*p* < 0.01, **A**) while the glycolytic metabolic potential was higher (*p* < 0.01, **B**). ASA had no effect in either strain (*p* > 0.05). No differences in coupling efficiency was observed between CON and *mdx* FDB fibres (*p* > 0.05, **C**). In CON fibres, ASA increased citrate synthase (CS) activity (*p* < 0.05, **D**) with ASA having no effect in *mdx* ASA fibres (*p* > 0.05). In both CON ASA and *mdx* ASA fibres, ASA increased the total mitochondrial pool compared to untreated fibres (*p* > 0.01, **E**). The mitochondrial viability was comparable in CON, CON ASA and *mdx* fibres (*p* > 0.05, **F**). ASA increased mitochondrial viability in *mdx* ASA compared to untreated *mdx* fibres (*p* > 0.05). In CON fibres, ASA increased O_2_^−^ production (*p* < 0.05, **G**) while in *mdx* fibres, ASA decreased O_2_^−^ production (*p* > 0.05). *n* = 9–11 for mitochondrial function and *n* = 3 for mitochondrial viability, pool and O_2_^−^ production CON, *n* = 9–10 for mitocho*n*drial function and *n* = 6–7 for mitochondrial viability, pool and O_2_^−^ production CON ASA, *n* = 10–14 for mitochondrial function and *n* = 6–10 for mitochondrial viability, pool and O_2_^−^ production *mdx*, *n* = 10–15 for mitochondrial function and *n* = 6–8 for mitochondrial viability, pool and O_2_^−^ production *mdx* ASA.

Using a mitochondrial stress test, we first determined the metabolic potential (corrected for basal respiration rate^39^) of the OCR and ECAR, which demonstrates the capacity of the metabolic pathways to ramp up in response to FCCP-induced mitochondrial uncoupling and depletion of the ∆Ψ for mitochondrial oxidative and cytosolic anaerobic metabolism, respectively. The oxidative metabolic potential, which assesses the capacity to potentiate oxidative metabolism during metabolic stress, was reduced by ~20% in *mdx* compared to CON FDB fibres (*p* < 0.01, Fig. 3A). In contrast, the glycolytic metabolic potential, which assesses the capacity to potentiate glycolysis during metabolic stress, was ~110% higher in *mdx* compared to CON FDB fibres (*p* < 0.01, Fig. 3B) demonstrating a shift toward a more anaerobic phenotype. The mitochondrial coupling efficiency, which indicates the extent to which ATP production at Complex V (CV) is matched to oxygen consumption at Complex IV (CIV), was comparable in CON and *mdx* FDB fibres (Fig. 3C). Contrary to our hypothesis that ASA would induce anaplerosis, there was no effect of treatment on either the oxidative or anaerobic metabolic potential, or the coupling efficiency, in CON or *mdx* FDB fibres. Of note, there was no ASA exogenously introduced into the assay, thus, the potentially short-lived effects of ASA may have been lost once the muscle was removed from the blood supply (and therefore the ASA supply) and incubated for 24 hours. To test this possibility, we next measured CS activity in FDB fibres that were snap frozen immediately after isolation, as an indicator of mitochondrial functional capacity (particularly that of Complex II (CII))^46,47^. While there was no difference in the CS activity of CON and *mdx* FDB fibres (Fig. 3D), ASA treatment did increase CS activity by 24% in CON fibres (*p* < 0.05) demonstrating anaplerotic capacity. Interestingly, ASA treatment had no effect on *mdx* fibres (*p* > 0.05), supporting our previous findings that *mdx* mitochondrial do not respond normally to metabolic stimulants^17^.

#### Mitochondrial Viability and Superoxide (O_2_^−^) Production

Since ASA was shown to stimulate CS activity only in CON muscle, we have investigated other potential effects of ASA at the mitochondrial level. First, we quantified the total mitochondrial pool (using MitoTracker green) and demonstrated that, while mitochondrial content is comparable between untreated CON and *mdx* fibres (Fig. 3E), ASA treatment was able to increase mitochondrial content in both strains by 55% and 208%, respectively (*p* < 0.01). Next, we assessed the viability of the mitochondrial pool by the ratio of live mitochondria (MitoTracker red) to the total mitochondrial pool (MitoTracker green). Mitochondrial pool viability was comparable in untreated CON and *mdx* FDB fibres (Fig. 3F). While ASA had no effect on mitochondrial pool viability in CON fibres, it did increase the viability of the mitochondrial pool in *mdx* fibres by 17% (*p* < 0.05, Fig. 3F).

Next, we assessed the effect of ASA on mitochondrial O_2_^−^ production to determine whether stimulating mitochondrial capacity merely resulted in enhanced reactive oxygen species (ROS) production, which could explain the lack of respiratory modulation. While there were no differences in the mitochondrial O_2_^−^ content of untreated CON and *mdx* fibres (Fig. 3G), ASA treatment increased O_2_^−^ production by 28% (*p* < 0.05) in CON fibres which was consistent with the stimulation of CS activity (24% increase). Although there was no anaplerotic effect of ASA treatment on CS activity, ASA decreased the O_2_^−^ content of *mdx* fibres by 26% (*p* < 0.05). Since ASA had no effect on mitochondrial coupling, these data suggest that in *mdx* muscle, ASA may induce an antioxidant response to improve the redox status of FDB fibres.

To interrogate the idea that ASA might modulate muscle redox status, we next assessed the relative time-course of changes in mitochondrial O_2_^−^ content in immortalised myoblasts derived from healthy CON and dystrophin-deficient DMD patients. Cells were treated with ASA for 24 hours, 3 days or 7 days and mitochondrial O_2_^−^ content relative to mitochondrial density was assessed using MitoSOX and MitoTracker Green dyes, respectively^36^. As described in Supplementary Fig. 3, 24-hour ASA treatment significantly increased mitochondrial O_2_^−^ content in DMD but not CON myoblasts. However, following 3 and 7 days treatment, ASA treatment significantly reduced the mitochondria O_2_^−^ content. These data support the notion of an ASA-dependent induction of the myofibre redox state.

#### Electron Transport Chain (ETC) Complex Expression

**Figure 4:**
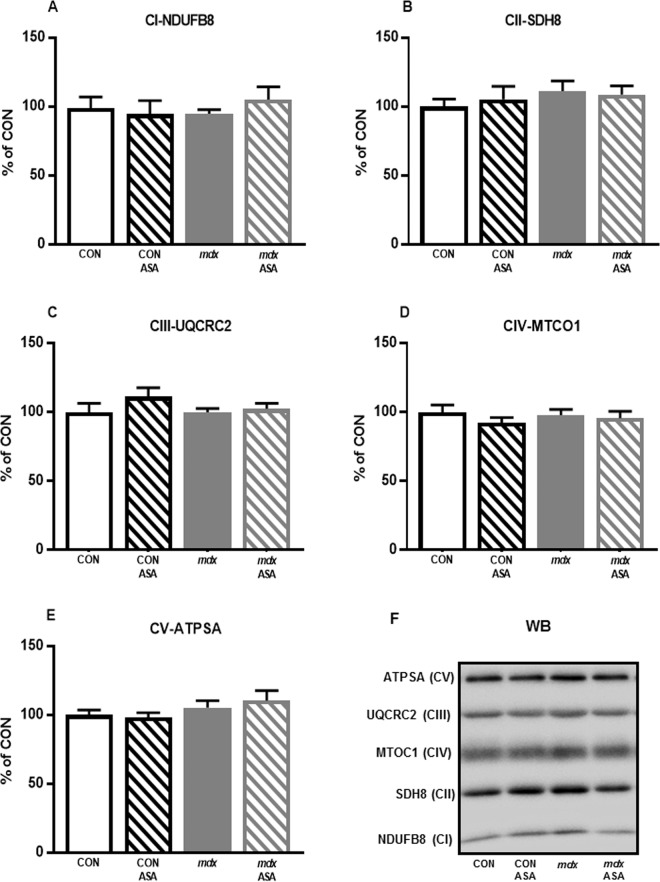
Mitochondrial respiratory chain complex proteins of the quadriceps from untreated and ASA-treated CON and *mdx* mice. No significant difference was detected between CON and *mdx* quadriceps in any subunit of the ETC complexes (*p* > 0.05, **A**–**E**). There was also no effect of ASA treatment (*p* > 0.05, **A**–**E**). Representative western blots of proteins from each of the five mitochondrial respiratory complexes (**F**). *n* = 8 per group.

The stimulation of mitochondrial biogenesis to enhance mitochondrial content without the concomitant upregulation of mitochondrial respiratory chain proteins to increase oxidative capacity is futile, and is characteristic of a fission phenotype in which ROS production replaces ATP production^48^. As such, we next assessed ETC complex expression to elucidate whether the capacity of ASA to enhance mitochondrial content translated to the modulation of respiratory chain density. As we did not have sufficient FDB fibres following respiratory analysis, we utilised the quadriceps for Western blot analysis due to a similar fibre type composition^49–52^. In all subunits quantified from CI to CV, no difference was detected between CON and *mdx* quadriceps (*p* > 0.05, Fig. 4). ASA had no effect on the expression of any of the mitochondrial complex subunits assessed in either strain which, together with an incapacity to modulate the oxidative potential, suggests that ASA induces fission (i.e. more, but smaller and less functional mitochondria) rather than biogenesis (i.e. more, fully-functional mitochondria that increase the overall respiratory capacity) in both strains, but more so in *mdx* muscles.

#### Metabolites

**Figure 5:**
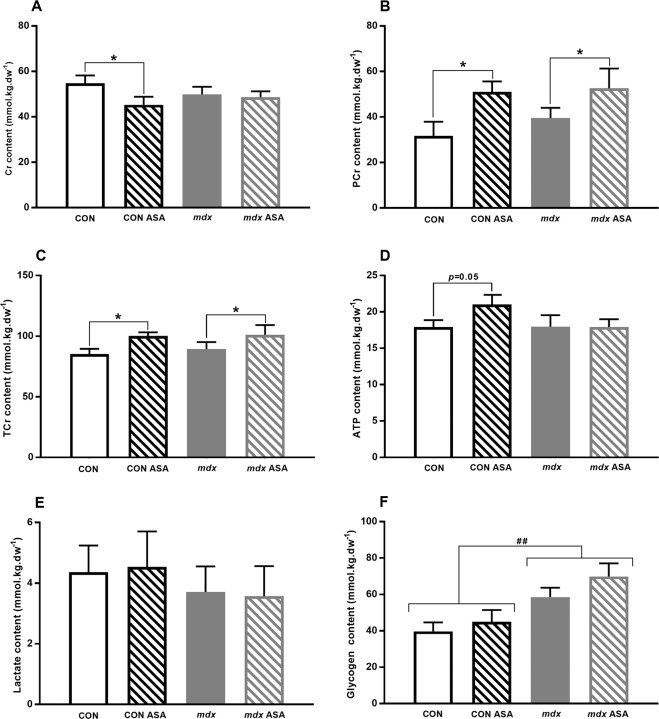
Intramuscular Cr, PCr, TCr, ATP, lactate and glycogen content of untreated and ASA-treated CON and *mdx* mice. No significant difference in Cr content was detected between CON and *mdx* TA (*p* > 0.05, **A**) with ASA increasing Cr content in CON ASA TA only (*p* < 0.05). While PCr content was comparable between CON and *mdx* TA (*p* > 0.05, **B**), ASA increased PCr content in both CON ASA and *mdx* ASA TA (*p* < 0.01). Similarly, although no genotypic differences were detected (*p* > 0.05, **C**), ASA increased TCr content in both CON ASA and *mdx* ASA TA (*p* < 0.05). ATP content was comparable between CON and *mdx* TA (*p* < 0.05, **D**) and there was a trend for ASA to increase ATP content in CON ASA only (*p* = 0.056). Lactate content was also comparable between CON and *mdx* TA (*p* > 0.05, **E**) and ASA had no effect. Overall, glycogen content was higher in *mdx* compared to CON (*p* < 0.01, **F**) TA and ASA had no effect in either genotype. Full-length blots are presented in Supplementary in Fig. 2. *n* = 6–12 CON, *n* = 8–14 CON ASA, *n* = 8–15 *mdx*, *n* = 8–15 *mdx* ASA.

To determine whether ASA could influence the overall metabolic signature of the skeletal muscle, we next evaluated the metabolite content of TA. In contrast to data described previously, the Cr, PCr, TCr, ATP and lactate content was comparable between untreated CON and *mdx* TA (Fig. 5A–E). While ASA had no effect on Cr content in *mdx* TA (Fig. 5A), ASA reduced the Cr content by 21% (*p* < 0.05) but increased intramuscular PCr content by 61%, in CON TA (*p* < 0.01, Fig. 5B), demonstrating enhanced high energy phosphate stores. The PCr content of *mdx* TA was also increased by 33% by ASA treatment (*p* < 0.01, Fig. 5B). As such, the TCr content was positively modulated by ASA treatment with a 17% and 13% increase in CON and *mdx* TA, respectively (*p* < 0.05, Fig. 5C). While ASA had no effect on the ATP content of *mdx* TA (Fig. 5D), there was a strong trend for ASA to increase ATP content in CON samples (*p* = 0.05). While, glycogen content was ~50% higher in *mdx* compared to CON TA (*p* < 0.01, Fig. 5F), consistent with an anaerobic phenotype, there was no effect of ASA on this metabolite in either strain.

#### Metabolic stress signalling

**Figure 6:**
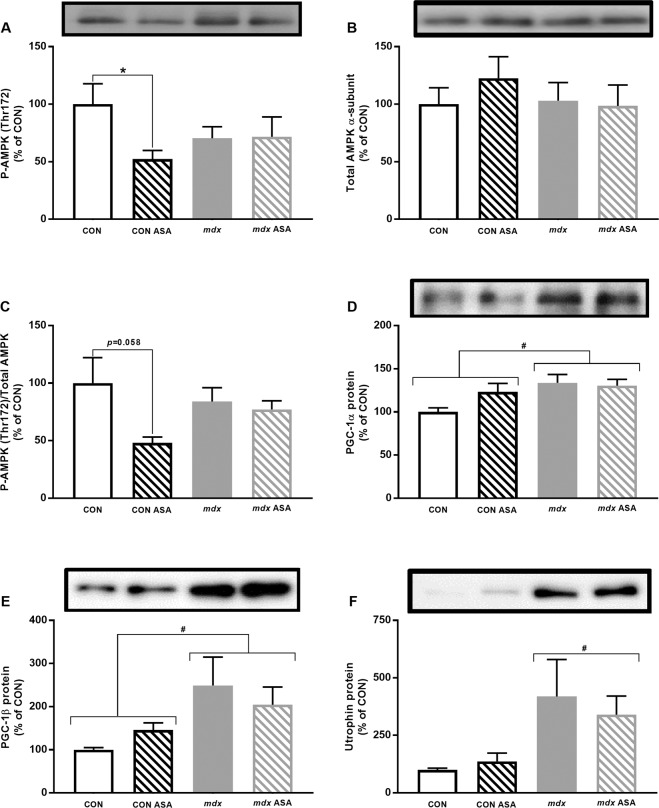
Metabolic signalling of untreated and ASA-treated CON and *mdx* mice. Phosphorylated AMPK (P-AMPK) was not different between CON and *mdx* quadriceps (*p* > 0.05, **A**) with ASA decreasing P-AMPK in CON ASA only (*p* < 0.05). There was no difference in Total AMPK (*p* > 0.05, **B**) detected between either strain or treatment regimen. Similarly, there was no difference in P-/Total AMPK ratio observed between CON and *mdx* TA (*p* > 0.05, **C**), however, while ASA had no effect on P-/Total AMPK ratio in *mdx* ASA quadriceps, there was a trend for ASA to decrease the ratio in CON ASA quadriceps (*p* = 0.058). PGC-1α and –β protein content was higher in *mdx* quadriceps compared to CON (*p* < 0.05, **D** and **E** respectively) with ASA having no effect (p < 0.05). Utrophin was elevated in *mdx* compared to CON TA (*p* < 0.05, **F**) and again, ASA had no effect in either strain. *n* = 7–8 CON, *n* = 6–8 CON ASA, *n* = 6–8 *mdx*, *n* = 6–7 *mdx* ASA.

During metabolic stress, when ATP cannot be resynthesised sufficiently to match metabolic demand, adenine nucleotides are rapidly degraded (ATP → ADP → AMP)^53^. Rising AMP levels activate AMP-activated protein kinase (AMPK) to modulate various responses to support ATP production, such as mitochondrial biogenesis, lipid metabolism and autophagy (as reviewed in^54^); thus preventing further breakdown of AMP to IMP. ASA supports the recovery/salvage of IMP to AMP, thus AMP-dependent metabolic adaptations might be a mechanism through which ASA exerts its therapeutic efficacy in dystrophin-deficient muscle. Phosphorylated AMPK (P-AMPK Thr^172^), a molecular marker of AMPK activation, was comparable between untreated CON and *mdx* quadriceps in our samples (Fig. 6A). Interestingly, and consistent with the ASA-induced increase in ATP content, ASA reduced P-AMPK in CON muscle only (by 47%; *p* < 0.05) but had no effect in *mdx* quadriceps. Total AMPK protein was also comparable between the strains, and ASA had no effect on this measure (Fig. 6B). While the ratio of P-AMPK to total AMPK was comparable between untreated CON and *mdx* quadriceps (Fig. 6C) and there was no effect of ASA treatment on *mdx* quadriceps, there was a strong trend for ASA to reduce this ratio in CON quadriceps (*p* = 0.058). Expression of the downstream regulators of mitochondrial biogenesis, PGC-1α and -1β, were elevated in *mdx* quadriceps by 34% and 149%, respectively, compared to CON quadriceps (*p* < 0.05, Fig. 6D,E respectively) with no effect of ASA. ASA thus appears to enhance metabolic stress response signalling (presumably by increasing AMP concentration) sufficient to increase ATP content (and presumably the ATP/AMP ratio) and decrease AMPK phosphorylation in CON muscle, but not in *mdx* muscle. This infers that the protective effect of ASA treatment on *mdx* histopathology is independent of AMPK modulation.

#### ASA does not modulate utrophin expression

To explore the potential that ASA might act as a genetic modifier of DMD due to the beneficial effects observed in histopathology, we assessed utrophin expression in TA. Utrophin expression is notably upregulated in the *mdx* mouse in the absence of dystrophin, resulting in a milder phenotype compared to humans^55^, and is induced by AMPK^56^. As expected, utrophin protein was ~4-fold higher in *mdx* TA (*p* < 0.05, Fig. 6E); however, ASA had no effect on utrophin expression in neither CON nor *mdx* TA.

## Discussion

While dystrophin-deficiency and sarcolemmal disturbance underpins DMD pathology, several lines of evidence highlight that metabolic insufficiency is a key aetiological modulator of disease progression (reviewed in^14^ and^19^). Considering the pivotal role that the mitochondria play in determining cell life and death, and the strong clinical data showing efficacy of the mitochondria-targeted CoQ10 analogue, idebenone, for attenuating respiratory decline in human DMD patients^18,57^, we investigated the potential of metabolic purine nucleotide therapy to ameliorate murine DMD. In particular, we examined the efficacy of the purine nucleotide ASA – which has anecdotally induced improvements in clinical indices of DMD progression^20^ – to pre-clinically re-evaluate it as a potential candidate for the treatment of DMD.

A striking finding in our study was that ASA therapy ameliorated key histopathological features of dystrophin-deficient muscle. Importantly, ASA reduced pseudohypertrophy, damage and, therefore, regenerative features (i.e. centronucleated fibres), fibrotic and lipid tissue infiltration, and Ca^2+^ content in *mdx* muscle. These histopathological features are most prominent as DMD advances^58–62^ and potentiate the deterioration of the functional capacity of skeletal muscles^63,64^. While we observed no improvements in tetanic force production following ASA treatment or in *mdx* compared to CON muscles, this finding is perhaps more reflective of limitations in the use of the *mdx* model rather than a lack of translatable functional improvement. Older (~100 day old) *mdx* mice have been previously shown to produce comparable forces to wild-type controls^65^ and reflects a regenerative capacity/stabilisation of the murine disease phenotype that is not observed in DMD patients. In this instance, an increase in fibre number and size functionally compensates for histopathological deterioration of the muscle. The reduced damage, and lipid and connective tissue infiltration following ASA treatment in our study highlights improvements in muscle integrity and quality that likely explain the maintenance of muscle strength and function observed in DMD and BMD patients treated with ASA previously^20^. Indeed, the same group has demonstrated excessive lipid production in explants of dystrophic muscle from DMD patients compared to healthy muscle cells^45^, which was also attenuated in the presence of ASA^45,66^. These data highlights translatable benefits of ASA for the treatment of DMD histopathology across murine and human species which apparently lead to functional improvements in human patients, whilst not necessarily in *mdx* mice. Notably, the ASA-dependent histopathological improvements observed in our study are independent of upregulated utrophin expression, the most well-established genetic modifier of murine DMD. While we cannot rule out the possibility of other genetic modifiers being influenced by ASA treatment, our data appears to consolidate a metabolic mode of action for ASA.

Metabolic dysfunction is a well-documented characteristic of dystrophic muscle, which is a contributing factor to disease progression (reviewed in^14^). We have demonstrated a reduced capacity of *mdx* muscle to utilise oxidative metabolism during metabolic stress (i.e. following FCCP-induced uncoupling in our experiments) albeit comparable ATP content in CON and *mdx* TA. Reduced ATP content has been widely reported in human DMD muscle (reviewed in^14^), but is difficult to accurately quantify given the transient nature of the molecule. A full metabolomics analysis would be required to ascertain the impact of reduced oxidative capacity on the metabolic systems, and the lack of one in this study is a limitation. Interestingly, our mitochondrial observations occurred despite increased PGC-1α (which has been previously observed^56^) and PGC-1β protein expression (reported for the first time here, albeit in quadriceps muscle). As there was no downstream increase in mitochondrial respiration, nor a difference in CS activity, SDH staining or mitochondrial density (assessed by MitoTracker Green) in untreated *mdx* compared to CON muscle, our data highlight that, despite an increased signal, dystrophin-deficient muscles do not adaptively expand the mitochondrial pool. While we did not observe differences in the expression of subunits of the ETC complexes between CON and *mdx* quadriceps (which would indicate a potential reason for the inability to increase ATP production), it is plausible that expression of other subunits of the ETC complexes are depressed or their activity is impaired. We have previously demonstrated that CI-mediated ATP production is severely impaired in *mdx* mitochondria irrespective of substrate supply, but that this can be attenuated with CI inhibition and stimulation of CII respiration^17^. When considered in context of our current data showing comparable mitochondrial content and expression of ETC subunits in *mdx* and CON muscle, these findings collectively suggest that functional impairments, possibly at CI, may be responsible for the impaired mitochondrial respiration. That being said, recent data has highlighted that immunoblotting may not be sensitive enough to detect small but physiologically relevant changes in protein expression at the mitochondrial level^67^. As such, structural impairments might also be accountable for our functional data, yet undetectable via Western blot.

Of note, *mdx* fibres appear to compensate for their reduced oxidative potential by increasing both their glycogen stores and their capacity for anaerobic glycolysis during metabolic stress. However, to what extent such compensation exists *in vivo* is unknown. While we have previously demonstrated that basal and exercise-induced glucose uptake are unaffected in *mdx* muscles^35^, enzymatic dysfunctions have been reported in the glycolytic pathway^68–71^ and could, therefore, limit the potential for anaerobic metabolism to buffer prolonged metabolic stress. Similarly, phosphorylase (the enzyme that breaks down glycogen) activity is reduced in dystrophin-deficient skeletal muscle^58,68–78^, thus it is unlikely that the compensatory upregulation of glycolytic flux could sustain metabolism *in vivo* in *mdx* mice. Certainly, when provided an opportunity for voluntary exercise, *mdx* mice are unable to run at the same speeds as their CON counterparts^15^.

We hypothesised that ASA could improve the metabolic capacity of dystrophic muscles by enhancing PNC function and thus purine nucleotide salvage, but particularly, by increasing fumarate production and anaplerosis of the mitochondrial TCA cycle^14,19^. Despite having previously demonstrated an impaired mitochondrial ATP production rate in isolated *mdx* mitochondrial bathed in optimal TCA substrate cocktails^17^, we and others have shown a partial attenuation of this mitochondrial dysfunction by stimulating CII with succinate^25–28,79–81^. Thus, we predicted in this study that promoting anaplerosis could, at the very least, augment SDH activity and the respiratory capacity of CII-mediated OXPHOS. However, our data demonstrate that ASA therapy is unable to modulate any of the mitochondrial parameters measured in FDB fibres in either CON or *mdx* mitochondria and is despite improving the viability and the density of the mitochondrial pool (as detected by MitoTracker dyes in dissociated FDB fibres). Our data was unexpected as the clinical trial of ASA in DMD and BMD patients anecdotally reported increases in stamina and energy levels while functional measurements were maintained^20^, which suggests that ASA can improve muscle fatigue properties. We did observe, however, that ASA induced changes in PCr content and the TCr pool suggesting manipulation of the Cr/PCr system to improve the energy buffering capacity and maintenance of ATP levels. Since TCr is the only metabolite measurement that is not influenced by the time-course of the experimental procedures (i.e. because it accounts for both high phosphate and degraded phosphate carrier, and, therefore, variations in the time taken to extract and snap freeze muscles) these are important data metabolically as it appears that some level of metabolic modulation is occurring in *mdx* muscle. The lack of effect of ASA at the mitochondrial level may be due to a few reasons. Firstly, as there is very limited literature regarding the human consumption of ASA, let alone as a metabolic therapy to treat neuromuscular disorders, it is unknown how long lasting the effects of ASA are. The improvements observed in the DMD and BMD patients in Bonsett and Rudman’s Phase I clinical trial were measured during the treatment period, which constituted infusion of ASA into the bloodstream via a miniature insulin pump^20^. In contrast, our mitochondrial measurements were made in FDB fibres removed from the *in vivo* (and ASA) environment some 24 hours earlier, thus the acute effects of ASA on the muscle – such as fumarate production and mitochondrial anaplerosis – may have been significantly dampened or completely lost due to the wash out of ASA. There is the potential that this purine metabolite has a short half-life and, therefore, any downstream anaplerotic effects on the TCA cycle and mitochondrial respiration may not be observed within our experimental timeframe (i.e. 24 hours post-tissue harvest). The fact that CS activity was assessed in snap frozen muscles and was significantly enhanced by ASA treatment in CON mice, certainly suggests that ASA therapy has an anaplerotic effect at the mitochondrial level. However, this effect was not observed in *mdx* muscles, highlighting the possibility of several genotype differences: (1) that ASA-induced anaplerosis cannot occur in dysfunctional mitochondria, perhaps due to the CI-defect purported by us previously^17^, or due to the malate dehydrogenase deficiency that has been reported by others^82^, resulting in substrate accumulation and inhibition of ATP synthesis; (2) that the conversion and transport of ASA-generated fumarate/malate via the malate-aspartate shuttle is defective; or (3) that ASA-generated fumarate is being sequestered away from the mitochondria into cytosolic reactions^83^.

The lack of obvious metabolic modulation by ASA appears to be specifically related to the *mdx* geno/pheno-type, since in CON muscles, ASA treatment did modulate some metabolic parameters. In particular, ASA increased ATP, reduced AMPK phosphorylation and apparently stimulated mitochondrial fission and anaplerosis as evidenced by increased MitoTracker green fluorescence without PCG-1α/β or ETC protein upregulation and increased CS activity, respectively. It has been postulated that a key role of mitochondrial fission is to prevent sustained mitochondrial elongation, which has been associated with the triggering of cellular senescence^84,85^ suggesting a cytoprotective role for ASA. Moreover, ASA altered the Cr/PCr system in CON mice which led to enhancement of the high energy phosphate pool (PCr and ATP). These data highlight beneficial effects, yet potentially differential mechanisms of action of ASA in CON and *mdx* mice. For CON mice, ASA-generated fumarate may be sequestered into the mitochondria, but without a heightened metabolic demand, anaplerosis seems to enhance O_2_^−^ as opposed to ATP production. This is presumably because: (1) ATP demand is being met; and (2) purine salvage is enhanced, thus alleviating the role of mitochondria in maintaining the bioenergetical status of the muscle. In contrast, ASA-generated fumarate may be directed into alternative reactions in *mdx* skeletal muscle to induce beneficial effects on dystropathology. While the mechanisms underlying the beneficial effects of ASA in *mdx* skeletal muscle remain unclear, ASA was shown to enhance mitochondrial viability and reduce O_2_^−^ production in *mdx* FDB muscles and in human DMD myoblasts after 3 and 7 days exposure. Oxidative stress is a well-documented feature of dystrophic muscle^8^, potentially due to the down-regulation of redox genes^86^. While we unexpectedly demonstrated comparable mitochondrial O_2_^−^ production between CON and *mdx* FDB fibres, the mitochondrial O_2_^−^ content of human DMD myoblasts was markedly higher than healthy controls at 24 hours and 7 days. FDB is a predominantly fast-twitch muscle and thus is not particularly dependent upon mitochondrial oxidative phosphorylation as a driver of ATP generation or likely a strong generator of ROS. Interestingly, ASA initially induced mitochondrial O_2_^−^ production in DMD myoblasts only and presumably reflects the metabolic stimulation of defective mitochondrial in which anaplerosis feeds ROS rather than ATP production. Whether it is the initial enhancement of ROS production or a direct modulation of the antioxidant response by ASA, which leads to modulation of the antioxidant response remains unclear. Nevertheless, the reduction of mitochondrial O_2_^−^ production via ASA is a positive finding in this study since radical production in dystrophin-deficient muscle is associated with enhanced muscle fibre damage and degeneration^8,87^. It has been previously demonstrated that fumarate content and cytosolic fumarase activity affects the redox status of the cell^88,89^. Increased fumarate content promotes glutathione production^88^ while the activity of cytosolic fumarase drives the malic enzyme reaction which generates NADPH^90^, thereby promoting the antioxidant status of the cell. Modulating cellular antioxidant systems such as that regulated by the glutathione system may be the mechanism through which ASA improves antioxidant defence in dystrophic muscle and, thus, warrants further investigation.

As this study is the first to experimentally evaluate ASA in a murine model, we closely monitored body weight and food and water consumption throughout treatment. No difference in weekly weight gain or food and water consumption was observed throughout the treatment period, although ASA did reduce the post-treatment body weights of *mdx* mice. While this was not reflected in differences in muscle weights relative to body weight, we did observe a decrease in lipid content of ASA treated *mdx* TA’s as detected by histology. Our data is consistent with a previous study that demonstrated reduced lipid production by DMD muscle explant cultures following ASA treatment^66^. The stimulation of β-fat oxidation may account for the reduced lipid content observed in our study, since it is plausible that lipid accumulation in dystrophic skeletal muscle results from a combination of enhanced lipid production^66^ and reduced fatty acid oxidation^91–94^. Once more, further investigation is required to elucidate the effect of ASA on body composition (i.e. via DEXA and/or micro-CT) and whether the modulation of lipid production compared to utilisation is a potential therapeutic mechanism of action.

## Conclusion

In summary, we are the first to demonstrate a remarkable capacity for ASA to ameliorate the histopathological hallmarks of murine DMD. This includes a reduction in intramuscular Ca^2+^ content, muscle damage and lipid and fibrotic tissue infiltration. ASA had no modulatory effect on *mdx* mitochondrial respiration although there was evidence of mitochondrial anaplerosis observed in healthy CON muscle, consistent with an underlying mitochondrial dysfunction in *mdx* muscles. Despite this, ASA reduced mitochondrial O_2_^−^ production, improved the density and viability of the mitochondrial pool and improved the overall metabolic signature of dystrophic *mdx* skeletal muscle. While we observed no functional (contractile force) improvements in *mdx* muscles following ASA treatment, we also saw no differences between CON and *mdx* muscles highlighting the limitations of the *mdx* mouse model at this age. Thus, future pre-clinical evaluation of ASA as a therapeutic candidate for DMD should utilise *mdx* mice undergoing active damage and associated loss of strength (i.e. at a young age (~28 days) or following exercise-induced damage). While the precise mechanism of action and a full characterisation of the antioxidant, cytoprotective and contractile function effects of ASA requires further elucidation, our data alongside anecdotal clinical observations in DMD patients, warrants further investigation of ASA as a therapeutic candidate for the treatment of DMD.

## Supplementary information

Supplementary information and figures.

## Data Availability

Data is available.
