# Supplementary material for: Adenylosuccinic acid therapy ameliorates murine Duchenne Muscular Dystrophy
Source: Sci Rep. 2020 Jan 24;10:1125. doi: 10.1038/s41598-020-57610-w (PMC6981178; doi:10.1038/s41598-020-57610-w)
Supplement: Supplementary file 1 — Supplementary information and figures. [file 41598_2020_57610_MOESM1_ESM.docx]

**Article Type**

Original Research Article

**Title**

Adenylosuccinic acid therapy ameliorates murine Duchenne Muscular Dystrophy

**Authors**

Cara A Timpani^1,2^, Craig A Goodman^1,2^, Christos G Stathis^1^, Jason D White^3,4^, Kamel Mamchaoui^5^, Gillian Butler-Browne^5^, Nuri Gueven^6^, Alan Hayes^1,2,7^, Emma Rybalka^1,2^

^1^Institute for Health and Sport, Victoria University, Melbourne, Victoria, 8001, Australia

^2^Australian Institute for Musculoskeletal Science (AIMSS), Victoria University, St Albans, Victoria, 3021 Australia

^3^Murdoch Children’s Research Institute, Royal Children’s Hospital, Parkville, Victoria, Australia

^4^Melbourne Veterinary School, University of Melbourne, Parkville, Victoria, Australia

^5^Institut de Myologie, Sorbonne University, INSERM UMRS974, Paris, France

^6^Pharmacy, School of Medicine, University of Tasmania, Hobart, Tasmania, 7000, Australia

^7^Department of Medicine-Western Health, The University of Melbourne, St Albans, Victoria, 3021, Australia

**Corresponding Author**

Dr Emma Rybalka

+ 61 3 83958226

emma.rybalka@vu.edu.au

**Running Head**

ASA therapy in the *mdx* mouse

**Materials and Methods**

**Cell Culture**

Dystrophin-positive (CON) muscle cells were derived from the paraspinal muscles of a 12 year old female and the dystrophin-negative (DMD) muscle cells derived from the fascia-lata of a 10 year old male with a deletion in exon 52 of the dystrophin gene.

Both CON and DMD cells were grown and maintained in growth medium containing low glucose 199 media (1:5, Gibco 11150059), high glucose Dulbecco’s modified eagle medium (DMEM) (4:5, Gibco 10566016), fetuin (25µg/mL; Sigma Aldrich F2379), human epidermal growth factor (5ng/mL; Sigma Aldrich E9644), basic human fibroblast growth factor (0.5ng/mL; Sigma Aldrich F0291), insulin (5µg/mL; Sigma Aldrich I5500), dexamethasone (0.2µg/mL; Sigma Aldrich D4902), gentamycin (50µg/mL; Gibco 15750060) and foetal bovine serum (20%; Bovogen Biologicals). Cells were seeded at a confluency of ~10% in a 75cm^2^ flask and passaged every 3 days. All cell experimentation was performed on undifferentiated myoblasts.

**Mitochondrial O_2_^-^ production**

CON and DMD myoblasts were seeded at a density of 5000 cells per well of a 96 well plate and treated with 1mM of ASA for either 24 hours, 3 or 7 days, with the media replaced every 3 days. Following the treatment period, mitochondrial O_2_^-^ production was quantified as described in “*Mitochondrial density, viability and superoxide (O_2_^-^) production*”.

**Supplementary Figures**


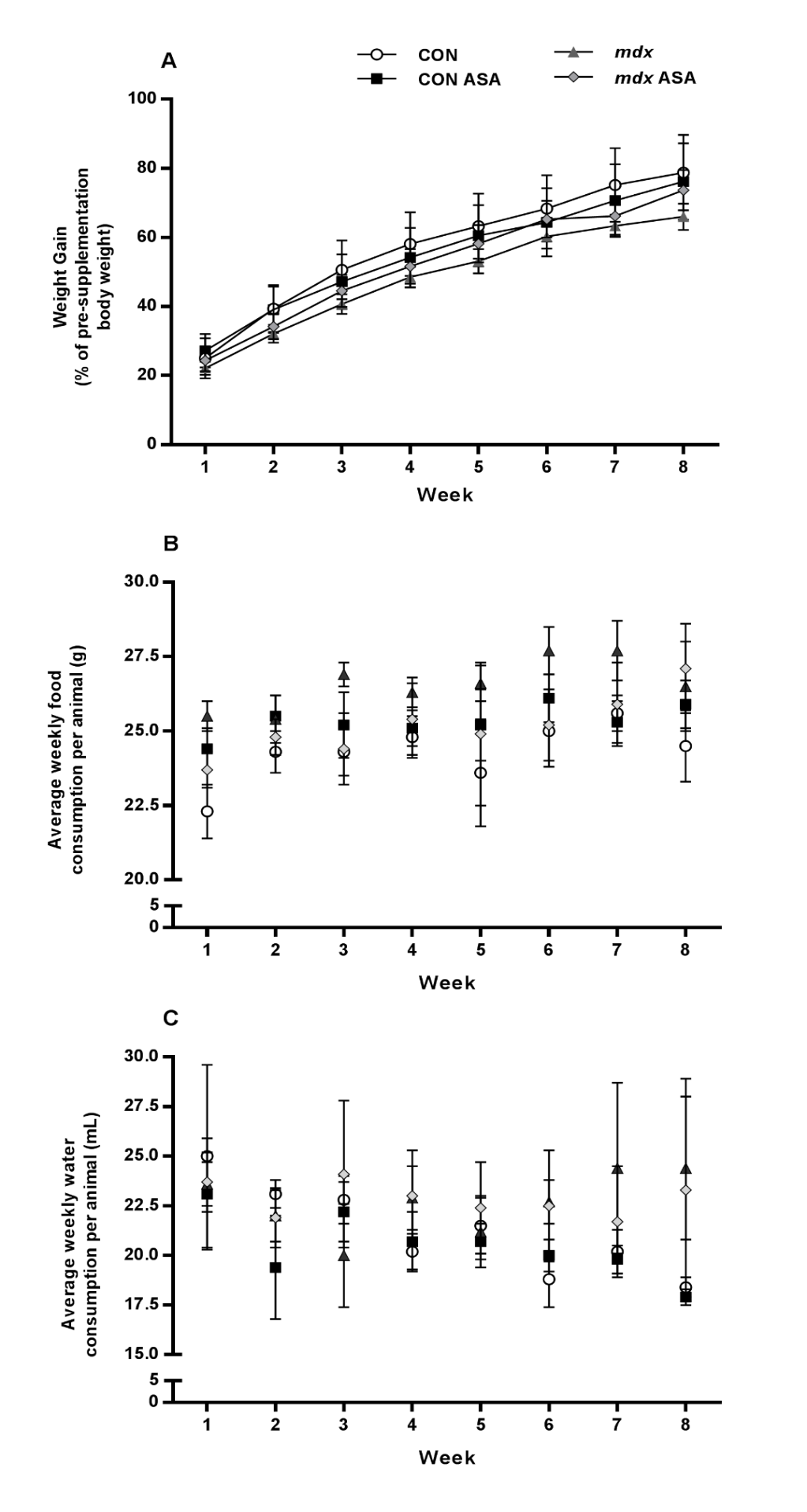


**Fig. S1 ASA treatment does not affect growth or food and water consumption of mice.** (A) Changes in body weight are shown as a percentage of pre-treatment weight. Over the 8 week treatment period, food (B) and water consumption (C) did not differ between untreated and treated animals (*p*>0.05). *n*= 16 per group.


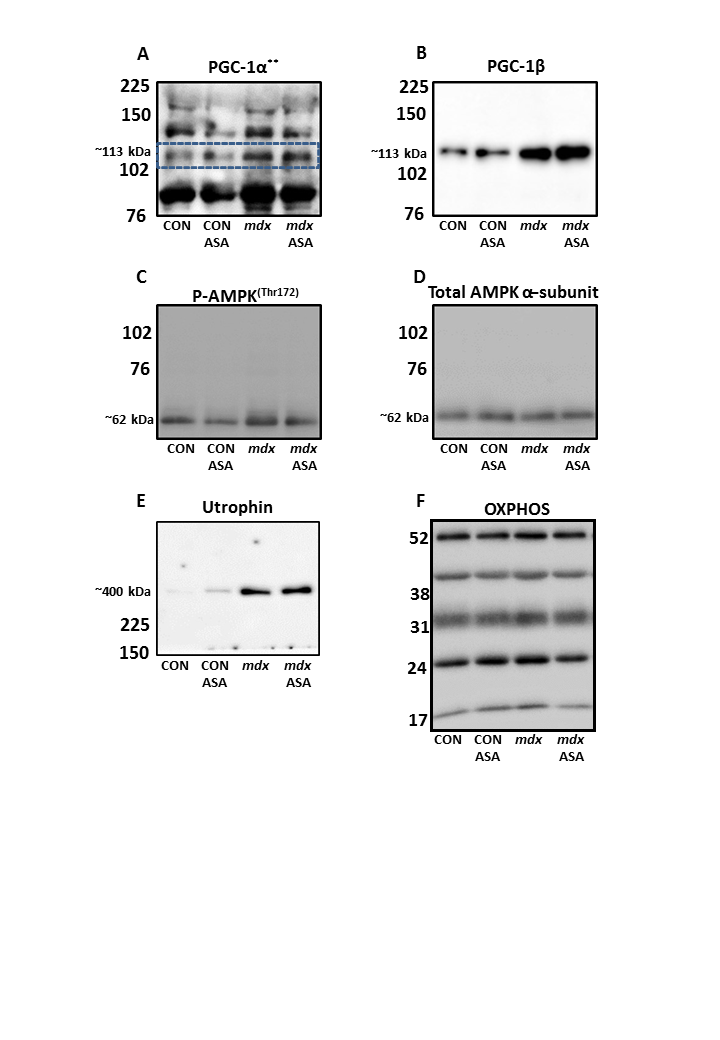


**Fig. S2 Full-length^1^ Western blot images relating to the data presented in Fig. 4 and 6.** ^1^After the transfer, membranes were cut horizontally to allow for probing with multiple primary antibodies on a single membrane/gel. Therefore, the above images display the largest available vertical membrane area probed with each respective primary antibody. ****** We have previously validated the ~113 kDa band detected by this primary antibody as PGC-1α. See Supplemental Figure 1 in [95].


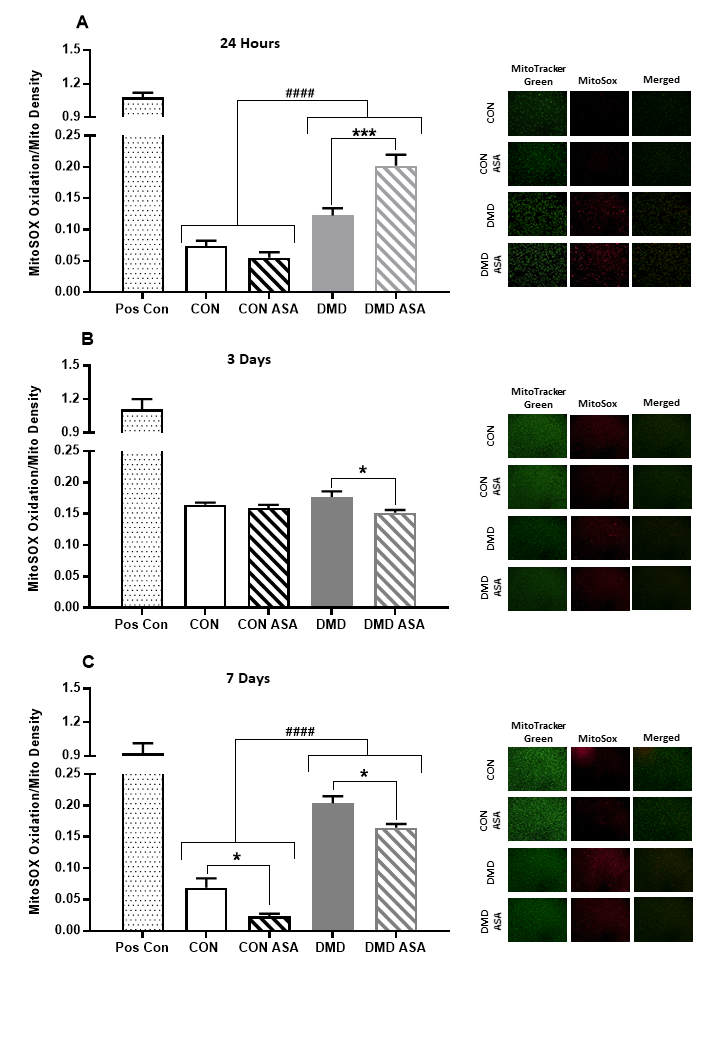


**Fig. S3 Mitochondrial superoxide (O_2_^-^) production of unsupplemented and adenylosuccinic acid (ASA) supplemented control (CON) and Duchenne Muscular Dystrophy (DMD) myoblasts.** Mitochondrial O_2_^-^ production was greater in DMD myoblasts compared to CON at 24 hours (*p*<0.0001, A) with ASA increasing O_2_^-^ production in DMD myoblasts only (*p*<0.001). At 3 days, mitochondrial O_2_^-^ production was comparable between CON and DMD myoblasts (*p*>0.05, B) with ASA supplementation decreasing O_2_^-^ production in DMD myoblasts (*p*<0.05). At 7 days, O_2_^-^ production was greater in DMD myoblasts compared to CON (*p*>0.0001, C) with ASA decreasing O_2_^-^ production in both CON and DMD myoblasts (*p*<0.05). *n*=4 per group.
